# Gene Expression Profiles of Sporadic Canine Hemangiosarcoma Are Uniquely Associated with Breed

**DOI:** 10.1371/journal.pone.0005549

**Published:** 2009-05-20

**Authors:** Beth A. Tamburini, Susan Trapp, Tzu Lip Phang, Jill T. Schappa, Lawrence E. Hunter, Jaime F. Modiano

**Affiliations:** 1 Integrated Department of Immunology, University of Colorado Denver, Denver, Colorado, United States of America; 2 Department of Pharmacology, University of Colorado Denver, Denver, Colorado, United States of America; 3 Department of Medicine, University of Colorado Denver, Denver, Colorado, United States of America; 4 University of Colorado Cancer Center, University of Colorado Denver, Aurora, Colorado, United States of America; 5 Department of Veterinary Clinical Sciences, University of Minnesota, Minneapolis and St. Paul, Minnesota, United States of America; 6 Masonic Cancer Center, University of Minnesota, Minneapolis, Minnesota, United States of America; Ohio State University Medical Center, United States of America

## Abstract

The role an individual's genetic background plays on phenotype and biological behavior of sporadic tumors remains incompletely understood. We showed previously that lymphomas from Golden Retrievers harbor defined, recurrent chromosomal aberrations that occur less frequently in lymphomas from other dog breeds, suggesting spontaneous canine tumors provide suitable models to define how heritable traits influence cancer genotypes. Here, we report a complementary approach using gene expression profiling in a naturally occurring endothelial sarcoma of dogs (hemangiosarcoma). Naturally occurring hemangiosarcomas of Golden Retrievers clustered separately from those of non-Golden Retrievers, with contributions from transcription factors, survival factors, and from pro-inflammatory and angiogenic genes, and which were exclusively present in hemangiosarcoma and not in other tumors or normal cells (*i.e.*, they were not due simply to variation in these genes among breeds). Vascular Endothelial Growth Factor Receptor 1 (VEGFR1) was among genes preferentially enriched within known pathways derived from gene set enrichment analysis when characterizing tumors from Golden Retrievers versus other breeds. Heightened VEGFR1 expression in these tumors also was apparent at the protein level and targeted inhibition of VEGFR1 increased proliferation of hemangiosarcoma cells derived from tumors of Golden Retrievers, but not from other breeds. Our results suggest heritable factors mold gene expression phenotypes, and consequently biological behavior in sporadic, naturally occurring tumors.

## Introduction

The role individual genetic backgrounds play on phenotypes and biological behavior of sporadic tumors remains to be determined in any species. Recent studies explored how race and ethnicity might influence gene expression and in turn contribute to disease susceptibility in humans, but few differences have been found [1], [2], [3]. Dog breeds may provide a useful surrogate for human ethnic groups. While dogs retain individual (outbred) traits, the derivation and maintenance of unique breeds has led to restricted gene pools. These restricted gene pools can be used to study heritable contributions to cancer susceptibility in animals that develop tumors spontaneously and share the human environment, but with the benefit of less “noise” from other phenotypic variation.

Recent work has emphasized the utility of spontaneous canine tumors as a robust, non-redundant model that complements studies in humans and laboratory animals to understand cancer genetics [4], [5]. For example, the degree of medical surveillance in dogs is second only to that in humans [6]; diseases such as cancer, where traits are genetically complex and whose prevalence increases with inbreeding, are well documented in dogs [5], [6]; and dog populations are structured into >400 partially inbred isolates (breeds) and a heterogeneous population of mixed-breed dogs. Gene flow between breeds is restricted by pedigree barriers and dogs of different breeds are often more (or less) susceptible to different diseases [5].

Equally important, the canine genome closely resembles the human genome, pet dogs share the human environment, and the lifetime cancer risk in dogs and humans is similar [7], [8]. Indeed, some cancers appear to occur more frequently in dogs and the incidence of many cancers varies according to breed, providing opportunities to study tumors that are difficult to replicate in humans or in inducible rodent models. Although cancer rates and incidence in dogs have not been established systematically in prospective or longitudinal studies, reproducible findings from retrospective analyses and breed health surveys provide reasonable estimates. Sporadic, naturally occurring hemangiosarcoma is relatively common in dogs (much more so than angiosarcoma in people, [9] with an apparent predilection for certain breeds such as German Shepherd Dogs, Boxers, and Golden Retrievers [9], [10], [11], [12], [13], [14]. The association between breed and disease is strengthened by information from recent breed health surveys. For example, cancer is the apparent cause of death for more than 60% of Golden Retrievers in the U.S. and the lifetime risks for any cancer, for hemangiosarcoma, and for non-Hodgkin lymphoma in this breed are 1 in 2, 1 in 5, and 1 in 8, respectively [15]. In contrast, the lifetime risk for any cancer and for hemangiosarcoma in Irish Setters are estimated at 1 in 3 and 1 in 34, respectively [16]. Other breed health surveys suggest hemangiosarcoma also is common in Portuguese Water Dogs and Australian Shepherds, whereas it is diagnosed less frequently in English Cocker Spaniels, Rottweilers, Gordon Setters, and Vizslas, among others.

Given the strong association between breed and risk, we predicted that gene expression profiles in tumors such as hemangiosarcoma also would reflect features uniquely associated with the breed. Furthermore, we anticipated that breed-related gene expression profiles would uncover biologically and therapeutically significant pathways that would inform etiology and identify therapeutic targets. Specifically, the central hypothesis was that naturally occurring hemangiosarcomas of Golden Retrievers would be distinguishable from histologically similar hemangiosarcomas of dogs from other breeds (non-Golden Retrievers) based on the overexpression or underexpression of genes preferentially concentrated in one or a few metabolic pathways, thus providing insights into the pathogenesis of this disease. To test this hypothesis, we used gene expression arrays and gene set enrichment analysis (GSEA) to identify genes that vary according to breed, as a proxy for heritability, in naturally occurring canine hemangiosarcoma. We hypothesized this would outline the potential influence of genetic background on cancer susceptibility and progression in a more unique way than simply comparing cancer cells to normal cells. For the first time, our data uncover unique gene sets that are peculiar to hemangiosarcoma tumors from a single dog breed (sharing a common genetic background). Overall, this study emphasizes the potential benefits of gene expression analysis and bioinformatics to study different biological aspects unique to a cancer susceptible dog breed and can fill gaps in our knowledge of disease susceptibility, heritability and progression.

## Results

### Gene Expression Analysis Segregates Canine Hemangiosarcoma According to Breed

While many human cancer cells have been shown to harbor different gene expression signatures compared to their normal counterpart cells (*e.g.*, [17]), little has been done to define gene expression profiles in canine tumors [18]. What is more, nothing has been done to outline how these phenotypes are influenced by heritable factors in any species. We showed elsewhere that hemangiosarcoma cells separate from non-malignant splenic hematoma cells based on gene expression profiles (Tamburini et al, manuscript in preparation). In this analysis, unsupervised clustering separated two major groups of hemangiosarcoma samples, consisting of tumors from Golden Retrievers and tumors from non-Golden Retrievers (GSE15086). Before we addressed potential differences in these two groups, however, we sought to ensure there were no hidden biases in the sample population. During the course of our study, we received blood samples from 76 dogs with pathologically confirmed hemangiosarcoma, including 48 Golden Retrievers and 28 non-Golden Retrievers. There were no differences between dogs in these two groups when comparing age at diagnosis (mean±S.D. = 9.3±2.6 and 8.6±2.6 years, respectively), gender (male vs. female, intact or neutered), location of the primary tumor, number of dogs treated, or outcome. The characteristics of the population were similar to those previously described both for Golden Retrievers [15] and for all dogs independent of breed [14], [19].

**Figure 1:**
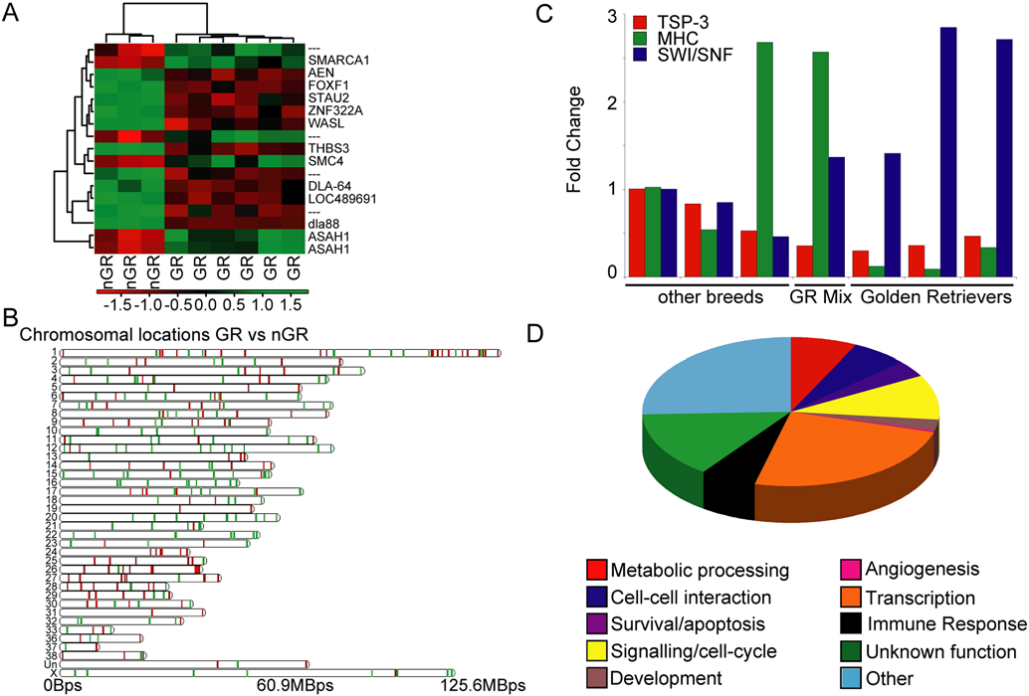
Golden Retriever Hemangiosarcoma Cells Segregate from Non-Golden Retriever Hemangiosarcoma Cells via Their Expression Profile. A. Hierarchical clustering of 6 Golden Retriever (GR) hemangiosarcoma samples versus 3 non-Golden Retriever (nGR) hemangiosarcoma samples (GEO series record GSE15086). Increasing green intensity indicates increased gene expression, increasing red intensity indicates decreased gene expression, and black indicates no change. Bars represent groups that cluster together. B. Gene differences between Golden Retrievers (GR) and non-Golden Retrievers (nGR) were plotted according to their cytogenetic location along the 38 autosomes and the X chromosome. Tick marks represent individual genes that show differential regulation, with the color intensity (green to black to red) representing expression changes as described in part A. Genes plotted received a p-value<0.05 and were derived from ANOVA analysis of the global list of filtered genes. C. Quantitative expression analysis of 3 genes found in panel A, TSP-3, DLA88 (MHC), and SMARCA1 (SWI/SNF) that were differentially expressed between Golden Retrievers with hemangiosarcoma and other breeds with hemangiosarcoma. Samples were evaluated for gene expression changes by RT-PCR followed by qPCR. One sample originating from a non Golden Retriever dog (Dal-4) was normalized to 1.0 and used as a reference; gene expression is presented as fold change compared to the reference sample. The samples used for real time PCR analysis (in the order presented) include CHAD P9, Dal-4, Joey, DD1, CHAD G6, CHAD G4, and Frog. D. Schematic representation of gene expression changes between Golden Retrievers and non Golden Retrievers with hemangiosarcoma grouped by biological function using ONTO/express gene ontology program (vortex.cs.wayne.edu/Projects.html).

Our gene profiling experiments included every sample for which viable tumor tissue was available to establish at least short-term cell cultures (N = 10, Table 1). The mean ages of the Golden Retrievers (N = 6) and non-Golden Retrievers (N = 3) in this subgroup were 10 and 8.3 years, respectively, while the latter group consisted only of male dogs. The final sample originated from a 9 year-old male Golden Retriever×Great Pyrenees F1 dog (F1). Age and gender as variables did not account for the observed clustering of the samples: when we segregated the 10 tumor samples into groups where affected dogs were younger than 7 years vs. older than 7 years or into male vs. female dogs, there were no significant differences in gene expression profiles. Nevertheless, a pattern remained when the nine tumor samples from purebred dogs (excluding the sample from the F1) separated according to breed. False Discovery Rate (FDR) analysis separated Golden Retriever and non-Golden Retriever samples into distinct groups with a 5-gene signature of MHC DLA88, Forkhead box protein F1, Thrombospondin-3 precursor, zinc finger protein 322A, and NAD(P) dependent steroid dehydrogenase. We anticipated that differences among hemangiosarcomas from dogs of different breeds would be subtle, thus the relatively small number of genes was not surprising given the relatively low expected discovery rate for this sample size. We took advantage of the predicted true positive rate and identified additional genes that were significantly different between the two groups at *p*<0.001. Figure 1A is a heat map illustrating hierarchical clusters defined by 12 known genes, 4 unknown genes and 1 repeated gene (acid ceramidase) isolated by two different probes. The list of known genes includes an additional MHC gene, genes involved in DNA replication and maintenance, and genes that regulate cellular metabolism (Table 2). When gene differences were plotted according to their cytogenetic location, there were few notable changes. Unlike the significant global underexpression seen when tumors were compared to non-malignant cells, samples from Golden Retrievers showed a net increase in the sum of expression of genes located in CFA 3, CFA 25 and CFA 30, and a net reduction in the sum of expression of genes located in CFA 12, CFA 14, CFA 29, CFA 32, CFA 33, and CFA 34. Predictably, since samples from Golden Retrievers included 3 females, this group also showed a net increase in the sum of expression of genes in the X chromosome. Figure 1B shows the location of individual genes that were recurrently and significantly overexpressed or underexpressed in the Golden Retriever samples.

**Table 1:** Signalment (Demographics) of Dogs in Study.

Sample ID	Diagnosis	Breed	Sex	Age
CHAD G4	Hemangiosarcoma	Golden Retriever	Male	10
CHAD G6	Hemangiosarcoma	Golden Retriever	Female	12
CHAD G8	Hemangiosarcoma	Golden Retriever	Male	12
FROG	Hemangiosarcoma	Golden Retriever	Female	10
JOURNEY	Hemangiosarcoma	Golden Retriever	Female	11
TUCKER	Hemangiosarcoma	Golden Retriever	Male	6
JOEY	Hemangiosarcoma	Rottweiler	Male	9
DD-1	Hemangiosarcoma	Golden Retriever×Great Pyrenees	Male	9
CHAD P9	Hemangiosarcoma	Portuguese Water Dog	Male	9
DAL-4	Hemangiosarcoma	Dalmatian	Male	7
FOREST	Unaffected	Golden Retriever	Male	10
HANK	Unaffected	Golden Retriever	Male	10
TUX	Unaffected	Golden Retriever	Male	10
JASPER	Unaffected	Boxer	Male	8
T	Unaffected	German Shorthair Pointer	Male	11
INGO	Unaffected	Rottweiler	Male	10
QUANTUM	Melanoma	Golden Retriever	Male	13
CHESTER	Melanoma	Golden Retriever	Male	14
REP	Melanoma	Miniature Schnauzer	Male	11
BAXTER L	Osteosarcoma	Golden Retriever	Male	8
JAZZ	Osteosarcoma	Golden Retriever	Male	7
KODIAK	Osteosarcoma	Great Pyrenees	Male	9
STRETCH	Osteosarcoma	Greyhound	Male	8.5
NELLIE	T-cell lymphoma	Golden Retriever	Female	6
PUEBLO	T-cell lymphoma	Golden Retriever	Male	11
MURPHY	T-cell lymphoma	Boxer	Male	9
RUFFIAN	B-cell lymphoma	Boykin Spaniel	Male	11

**Table 2:** Gene Expression Analysis Separates Golden Retriever Hemangiosarcoma Tumors from Non-Golden Retriever Hemangiosarcoma Tumors^1^.

Gene title	Chrom.	Function	Fold Change	p-value
similar to N-acylsphingosine amidohydrolase (acid ceramidase) 1	16	Metabolic processing	1.79	3.6E-04
MHC class 1 DLA-88	12	Cell-cell interaction	−603.8	1.7E-08
similar to Thrombospondin-3 precursor	7	Cell-cell interaction	−2.08	1.1E-04
similar to NAD(P) dependent steroid dehydrogenase-like	5	Cell-cell interaction	−1.91	1.7E-04
MHC class 1 DLA-64	12	Cell-cell interaction	−2.81	6.9E-04
similar to Wiskott-Aldrich syndrome gene-like protein	14	Cell-cell interaction	−1.60	7.4E-04
similar to staufen, RNA binding protein, homolog2 isoform LL (A)	29	Cell-cell interaction	−2.02	8.2E-04
MHC class 1 DLA-88	12	Survival/apoptosis	−603.8	1.7E-08
similar to interferon stimulated exonuclease gene 29 kDa-like 1	3	Survival/apoptosis	−2.23	5.1E-04
MHC class 1 DLA-64	12	Surivival/apoptosis	−2.81	6.9E-04
similar to SWI/SNF-related matrix associated actin-dependent regulator of chromatin remodeling	X	Signaling/cell cycle	3.42	5.7E-04
similar to Structural maintenance of chromosomes4-like 1 protein	34	Signaling/cell cycle	1.72	6.8E-04
similar to Forkhead box protein F1	5	Transcription	−1.65	9.3E-05
similar to zinc finger protein 322A	35	Transcription	−1.68	1.5E-04
similar to SWI/SNF-related matrix associated actin-dependent regulator of chromatin remodeling	X	Transcription	3.42	5.7E-04
MHC class 1 DLA-88	12	Immune response	−603.8	1.7E-08
similar to interferon stimulated exonuclease gene 29 kDa-like 1	3	Immune response	−2.23	5.1E-04
MHC class 1 DLA-64	12	Immune response	−2.81	6.9E-04
Transcribed locus [Cfa.6637.1.A1_at]	9	Unknown	−6.10	5.3E-04
Transcribed locus [Cfa.14890.1.A1_at]	28	Unknown	2.82	9.8E-04
— [CfaAffx.1401.1.S1_at]	1	Unknown	−1.59	8.4E-04
— [Cfa.11358.1.A1_at]	16	Unknown	2.02	1.0E-03

1 The list represents genes that were significant to p<0.001 comparing tumors from Golden Retriever to tumors from non-Golden Retriever. Each gene is grouped into functional categories as defined in Fig. 1D. Mean fold change reflects the average expression in cells from Golden Retriever tumors over the average expression in cells from tumors of non-Golden Retrievers; p-values were calculated after verifying the data were normally distributed using Student's T-test. Some genes are found within multiple functional categories.

We used reverse transcriptase PCR followed by quantitative real time PCR analysis of DLA-88 (MHC), TSP-3 and SMARCA-1 (SWI/SNF) expression to verify the microarray data (Figure 1C). We included each of the non-Golden Retrievers (Dal-4, Joey, and CHAD-P9) and three Golden Retrievers (CHAD G6, CHAD G4, and Frog) for this analysis. The genes were chosen because they may define MHC haplotypes or because of their relevance to tumor biology; *i.e.*, DLA-88 is an MHC class I gene [20], homologues of TSP-3 are known to regulate angiogenesis [21], and the SWI/SNF related gene SMARCA-1 is an ATP dependent chromatin remodeler important for the regulation of transcription, DNA replication, and DNA repair that is abnormally expressed in certain tumors [22]. Figure 1C and Table 2 show TSP-3 and DLA-88 were consistently underexpressed, whereas SMARCA-1 was consistently overexpressed in hemangiosarcomas from Golden Retrievers. This latter gene is encoded in the X chromosome, but the data suggest this is not purely a female bias: SMARCA1 expression was actually highest in cells from CHAD G4, which was a male dog (Figure 1C, middle dog in the Golden Retriever group). Student's T-test for equal variance was used to calculate p-values as an indication of statistical significance (Table 2). The availability of a sample originating from an F1 mix-breed dog with fortuitously known parentage (Golden Retriever×Great Pyrenees) allowed us to ask interesting, albeit anecdotal questions. Specifically, was this dog more similar to Golden Retrievers, to non-Golden Retrievers, or would it reflect a “mixture” of both? When we included this sample in the hierarchical clustering, the features that separated the two groups were less distinguishable. Only 7 of the 17 signals on the hit list remained among the 35 genes with lowest p-values (p<0.0123) and 11 of 17 were found in the top 200 (p<0.04). This suggested that “Golden Retriever” contributed, but did not completely control the gene expression signature in this F1 dog's tumor. Figure 1C shows indeed, that expression of TSP-3 in the F1 (Golden Retriever mix) was similar to the Golden Retriever group and expression of MHC DLA-88 was similar to the non-Golden Retriever group. Thus, the expression of genes in the tumor was predictably modulated by the dog's Golden Retriever and non-Golden Retriever background.

One possible explanation for why Golden Retrievers separate from non-Golden Retrievers in this analysis is that hierarchical clustering by breed reflected unique properties of genetic variants within the breed, rather than a particular influence of breed on tumor phenotypes. To our knowledge, there is no reported association between breed and MHC haplotypes, so this was unlikely. Nevertheless, we examined whether the association between expression of TSP-3, DLA-88, or SMARCA-1 and breed (Golden Retriever) would hold in non-hemangiosarcoma samples. Samples analyzed included blood leukocytes from healthy Golden Retrievers and non-Golden Retrievers, blood leukocytes from Golden Retrievers and non-Golden Retrievers that did not have hemangiosarcoma, but were diagnosed with another cancer (melanoma, non-Hodgkin lymphoma, or osteosarcoma), and the hemangiosarcoma cells from each affected dog (Table 3). In blood samples from healthy dogs and dogs with other types of cancers, expression of TSP-3, MHC DLA-88, or SWI/SNF (SMARCA1) was not significantly different among groups. However, in hemangiosarcoma samples from Golden Retrievers, the expression of TSP-3 and DLA-88 was consistently lower, and the expression of SMARCA1 was consistently higher than in non-Golden Retrievers (p<0.03). One interesting observation is that the range of expression for these genes in the hemangiosarcoma samples and in blood samples from healthy dogs were narrow, but they were relatively wide in blood samples from dogs that had non-hemangiosarcoma tumors. Even so, the trends for expression of TSP-3 and DLA-88 are reversed in these samples. This suggests the differences were not due to variants in the breed, and instead were due to the influence of genetic background (breed) itself on hemangiosarcoma phenotypes. Another possibility was that this difference would be reflected only on tumor samples, so we assessed whether these genes had significantly different calls when comparing our hemangiosarcoma Golden Retriever expression arrays to expression arrays from lymphoma and leukemia (30 Golden Retrievers) and from osteosarcoma (9 Golden Retrievers). The association between hemangiosarcoma and overexpression of acid ceramidase was reinforced in these analyses, but neither TSP-3, nor DLA-88, nor SMARCA1 showed differential expression according to breed in lymphoma and leukemia or in osteosarcoma, although those samples also appear to have different and unique sets of genes whose expression varies as a function of breed (T. Phang, K. Gavin, A. Sarver, and J. Modiano, unpublished data).

**Table 3:** Breed-Dependent Gene Expression Differences in Hemangiosarcoma Are Not Generalized To Normal Tissues or Other Tumors^1^.

Tissue Type	Average fold change of TSP-3 GR vs nGR (Mean [Range])	p-value	Average fold change of MHC GR vs nGR (Mean [Range])	p-value	Average fold change SWI/SNF (SMARCA1) GR vs nGR (Mean [Range])	p-value
Hemangio-sarcoma (tumor)	0.47 [0.39–0.56]	0.025	0.16 [0.07–0.26]	0.029	3.18 [2.04–4.32]	0.028
Healthy (blood)	0.84 [0.52–1.16]	0.623	1.14 [0.98–1.30]	0.783	0.78 [0.40–1.15]	0.571
Tumors (blood)	3.95 [0.82–7.08]	0.558	6.93 [2.49–11.37]	0.279	22.60 [1.98–43.22]	0.174

1 qPCR was performed on genes as described previously in Figure 1C. Presented is the average fold change and average fold range from at least 3 samples which were individually normalized to 18s control gene. P-values were calculated using the Welch t-test for samples with unequal variance, or Student's t-test for equal variance. Only Golden Retriever hemangiosarcoma compared to Non-Golden Retriever hemangiosarcoma showed differences that were statistically significant in each of the 3 genes analyzed.

### Pathway Analysis Provides Insight into Hemangiosarcoma Susceptibility and Heritability

**Figure 2:**
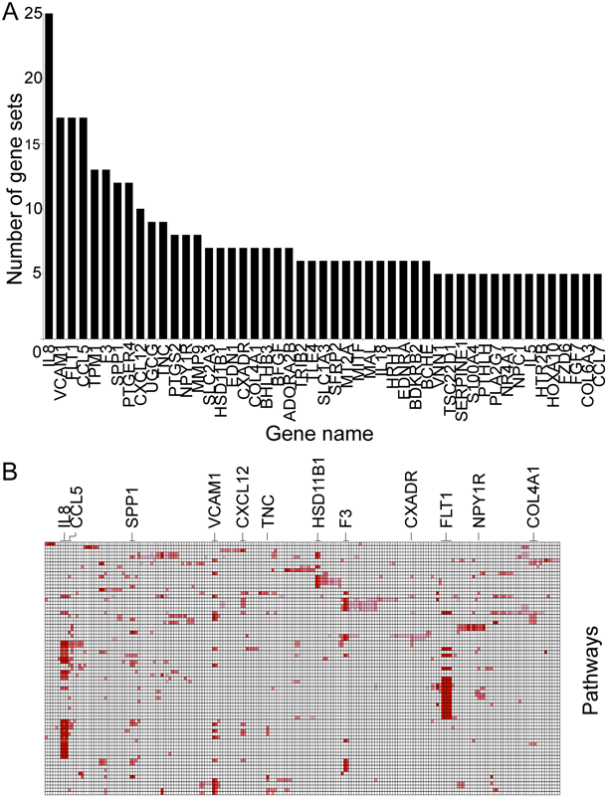
Gene Set Enrichment Analysis Validates the Hypothesis that the Golden Retriever Hemangiosarcoma Gene Set Is Involved in Hypoxia, Inflammation, and Cancer. A. Bar graph representing the number of gene sets/pathways from the GSEA archived database that were enriched in hemangiosarcoma samples from Golden Retrievers versus hemangiosarcoma samples from non-Golden Retrievers. Each gene on the x-axis was present in the number of GSEA gene sets indicated on the y-axis (of 77 where FDR<0.05). B. Graphical representation of genes (x-axis) present in each GSEA pathway/gene set (y-axis), where a filled box means the gene was present and enriched in that GSEA pathway. Increasing red intensity reflects higher enrichment scores. The genes enriched in the highest number of gene sets are identified by name.

**Figure 3:**
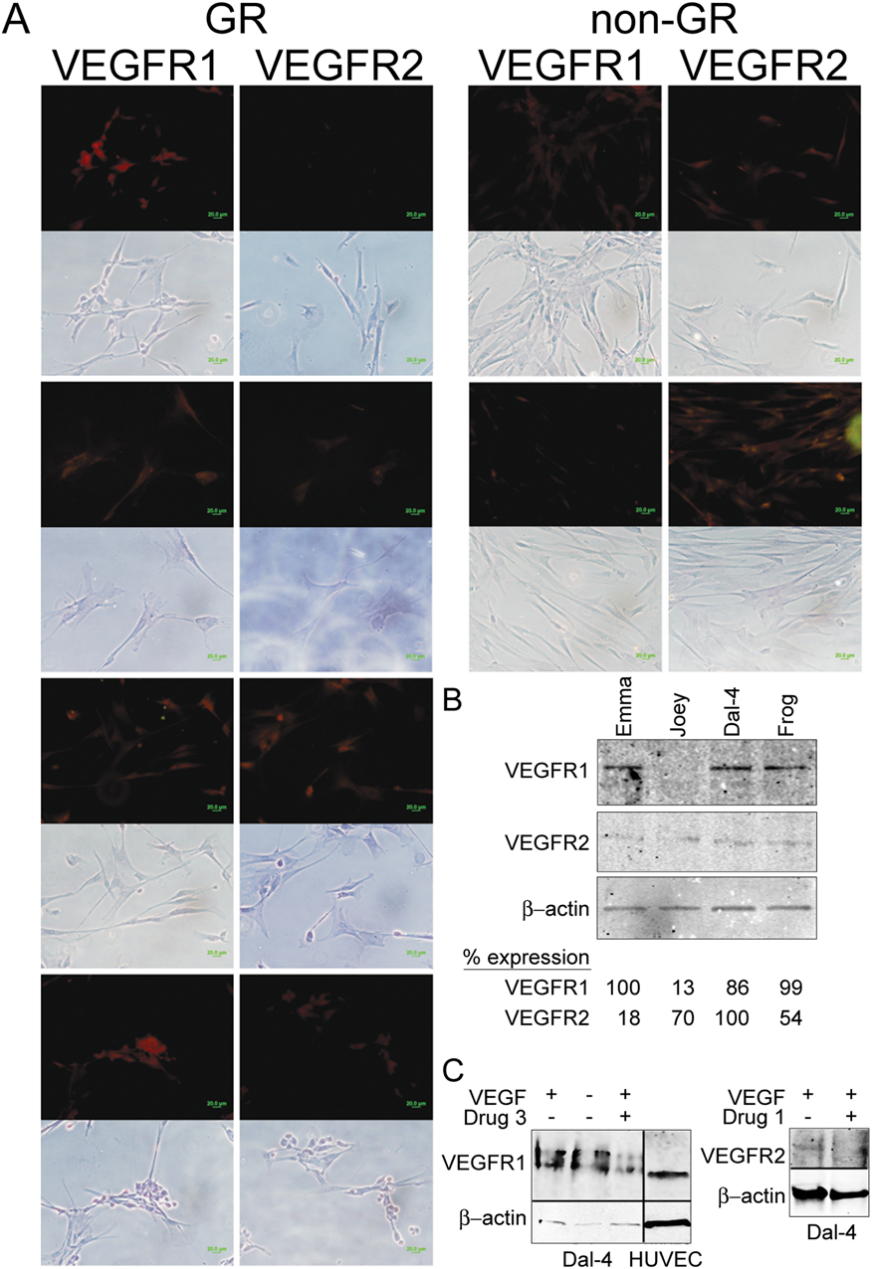
Expression of VEGF Receptors in Hemangiosarcoma Cells of Golden Retrievers and Non-Golden Retrievers. A. Hemangiosarcoma cells from 4 Golden Retrievers (in order from top to bottom, Frog, Veronica, Tucker, Emma) and from 2 non-Golden Retrievers (Dal-4 and Joey) were cultured in chamber slides and stained with antibodies against VEGFR1 and VEGFR2 as described in the methods. Staining was visualized using epifluorescence. Bar = 20 µm. B. Emma, Frog, Joey, and Dal-4 cells obtained during the log growth phase were used to quantify expression of VEGFR1, VEGFR2 and ß-actin by immunoblotting. Conditions were optimized for linearity. Densitometric band quantification was done using Image J 1.37. Data are normalized to ß-actin using the sample with the highest expression for each receptor as the calibrator. C. Dal-4 cells were cultured in complete media supplemented with VEGF (+), in the absence of serum and growth supplements (−), with or without Drug 3 (100 nM) or Drug 1 (1 µM) as indicated. The activation status of VEGFR1 and VEGFR2 was examined using modification-state (phosphospecific) antibodies directed against pVEGFR1-Tyr915 and pVEGFR2-Tyr875. ß-actin was used as a loading control, and HUVEC lysates were used as a specificity control for VEGFR1.

When we compared tumors from Golden Retrievers against tumors from non-Golden Retrievers with hemangiosarcoma, we found differentially expressed genes in several functional categories defined by ONTO/express (Figure 1D). The single largest category where genes differed between the two groups was genes involved in transcription. We then applied GSEA to improve the definition of pathways that may be influenced by heritable traits and identified 77 gene sets with FDR<0.05 (Table S1). GSEA is designed to identify categories, families, or sets of genes where there are potentially small but coordinated changes in gene expression. In other words, the intent was to discover groups of genes (annotated by pathway) that “move” as a group, but where the separation of any individual gene in the group would not be, by itself, necessarily statistically significant. The top gene sets identified with FDR<0.001 and with normalized enrichment scores (NES)<2.1 are shown in Table 4. GSEA highlighted unique differences between hemangiosarcomas segregated by breed: for example, Flt-1/VEGFR1 was exclusively enriched in GSEA pathways separated according to breed (Figure 2). The enrichment of VEGFR1 in these cells was especially intriguing. Previous flow cytometric and immunocytochemical analysis of hemangiosarcoma samples from Golden Retrievers and from non-Golden Retrievers showed expression of levels of CD133, CD34, c-Kit, CD45, CD146, and α_v_ß_3_-integrin [23], [24] were equivalent. Yet, immunologic analysis verified the GSEA data. Figure 3A shows immunocytochemical staining and immunoblotting for VEGFR1 and VEGFR2 in cell lines derived from Golden Retrievers and from non-Golden Retrievers. One recently developed line that had not been arrayed (Emma) was included as a means to provide validation of the data. Immunocytochemical staining verified each of the Golden Retriever-derived cell lines expressed VEGFR1. The relative expression of this protein as determined by immunoblotting was higher in Emma and Frog (Golden Retriever) cell lines than it was in Joey and in Dal-4 (non-Golden Retriever) cell lines, and conversely, the relative expression of VEGFR2 was higher in Joey and Dal-4 than it was in Emma and Frog (Figure 3B).

**Table 4:** Gene Set Enrichment Analysis Predicts Pathways Involved in Inflammation, Cancer, and Hypoxia Are Important for Golden Retrievers with Hemangiosarcoma^1^.

Gene set	Description	ES	NES	FDR
TARTE_MATURE_PC	Genes overexpressed in polyclonal plasmablastic cells	0.71	2.63	<0.001
IDX_TSA_DN_CLUSTER3	Genes downregulated during differentiation of 3T3-L1 fibroblasts into adipocytes	0.82	2.40	<0.001
CARIES_PULP_UP	Genes upregulated in pulpal tissue from extracted cavities	0.73	2.26	<0.001
HYPOXIA_REVIEW	Genes known to be induced by hypoxia	0.67	2.26	<0.001
RUTELLA_HEPATGFSNDCS_UP	Genes upregulated by hepatocyte growth factor treatment	0.70	2.19	<0.001
NAKAJIMA_MCS_UP	Most increased transcripts in activated human and mouse mast cells	0.77	2.14	0.001
TPA_SENS_EARLY_DN	Downregulated by TPA at two consecutive timepoints between 15 min–3 hrs in sensitive HL-60 cells	0.69	2.13	0.001

1 The filtered gene list from Golden Retrievers with hemangiosarcoma vs. non-Golden Retrievers with hemangiosarcoma were compared using the GSEA software. ES (Enrichment Score) is a value that represents how well the gene set is enriched within the selected gene list. NES (normalized enrichment score) corrects the ES for differences in gene set size and can be used to compare across gene sets. A high ES or NES indicates that gene set is highly enriched within our gene list. FDR represents the probability that the NES for a gene set gives a false positive finding. The highest FDR shown here is 0.005 indicating that there is a 0.005% chance that the gene set indicates a false positive finding. The lists shown are those gene sets with an NES higher than 2.10.

**Figure 4:**
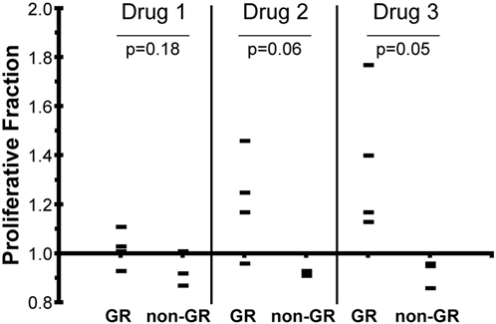
Differential Sensitivity of Canine Hemangiosarcoma Cell Lines to VEGFR Inhibitors. The effect of three VEGFR inhibitors on proliferation and viability of hemangiosarcoma cells was tested in vitro. The selectivity and half maximal inhibitory concentrations for Drugs 1, 2, and 3 are listed in the Materials and Methods. Cells (10,000/well) were plated in duplicate in a 96-well microtiter plates and allowed to attach for 16 hr prior to addition of inhibitors at the indicated concentration. Cells were then cultured for 72 hr, and the number of viable cells was determined using the MTS assay. Absorption at 490 nm for each well was averaged, and data normalized to % viability where the mean of wells that received no treatment (0 nm) was considered = 100%. The mean of two independent experiments is shown at drug concentrations of 100 nM. P-values were calculated using Student's T-test.

Finally, we examined if these expression patterns had functional correlates. We hypothesized that hemangiosarcoma cell lines from Golden Retrievers and from non-Golden Retrievers would show differential sensitivity to small molecules that selectively inhibit VEGFR1 and VEGFR2 kinase activity. We selected two compounds, referred to as “Drug 1” and “Drug 3” for simplicity, with distinct affinity for VEGFR1 and VEGFR2. Drug 1 is a selective VEGFR2 inhibitor, and Drug 3 is a related small molecule with similar affinity for VEGFR2 as Drug 1, but with 100-fold greater affinity for VEGFR1. Figure 3C illustrates a representative experiment that shows the VEGFR inhibitors we selected had the predicted effects to inhibit activation of each receptor in Dal-4 cells (one of the cell lines that had detectable VEGFR1 and VEGFR2), as determined by the steady state level of activating tyrosine phosphorylation at the residues homologous to human Tyr1213 in VEGFR1 and Tyr951 in VEGFR2. As would be predicted from the data in Figures 3A and 3B, we noticed some variation in the levels of phosphorylated VEGF receptors in the cells, mostly related to the overall steady state expression of these proteins. Figure 4 shows that Drug 1 did not significantly affect any of the seven cell lines tested. In contrast, cell lines derived from Golden Retrievers showed significantly greater proliferation in the presence of Drug 3 (Veronica>Tucker>Emma>Frog). These responses were dose dependent and peaked at concentrations of 0.1 to 10 nM. Drug 2, which has lower affinity for both receptors, did not significantly alter proliferation of hemangiosarcoma cells, but it is compelling that there was a trend for greater proliferation by the Golden Retriever tumor lines at higher concentrations (1 to 100 nM). Additionally, VEGFR1 did not appear over-represented in any of the other tumor types we examined from Golden Retrievers suggesting these changes are specific to Golden Retrievers with hemangiosarcoma. Together, the data indicate that gene expression patterns identified by gene set enrichment analysis across distinct subgroups are biologically significant, and in this case, they suggest VEGFR1 is not a decoy receptor, but rather it is an active growth inhibitor in hemangiosarcoma cells derived from Golden Retrievers.

## Discussion

The relevance of naturally occurring canine tumors to improve our understanding of cancer biology and genetics has been increasingly recognized in recent years [4], [5], [25]. Canine tumors can be utilized as a system to understand how genetic background can influence the susceptibility of an individual to non-inherited cancers. Due to the homogeneity among dog breeds, we can study frequently occurring cancers within groups in a way that would be difficult within the genetically diverse human population or in laboratory animals, where most tumors are induced chemically or by genetic manipulation.

We studied naturally occurring canine hemangiosarcoma to test the hypothesis that patterns of gene expression could outline biological differences between tumor cells originating from dogs of a distinct breed that have a higher lifetime risk for hemangiosarcoma. Hemangiosarcoma is ontogenetically related to human angiosarcoma and Kaposi sarcoma, as all three are presumed to arise from hemangioblastic or endothelial progenitors and they share signaling abnormalities [19], [23], [26]. The highly metastatic behavior and modest response to chemotherapy distinguish canine hemangiosarcoma and human angiosarcoma from other common soft tissue sarcomas that are locally invasive and generally unresponsive to chemotherapy. We uncovered a set of hemangiosarcoma-associated genes peculiar to a single dog breed suggesting these are modulated by (or with) heritable traits that may influence risk for this cancer.

We considered carefully the choice of low passage cell lines vs. intact tumors for these experiments. Tumors are in essence tissues [27]. Tumor cells modify the microenvironment and are themselves responsive to environmental cues. Nevertheless, to understand the contribution of the tumor cells to biological and pathological processes, it is important to be able to examine the response on isolated cells. One approach to do this is microdissection, but in a vascular tumor, it is difficult to microdissect malignant tissue without retaining normal angiogenic components, which are morphologically indistinguishable in many cases, and blood elements. On the other hand, cell lines provide a homogeneous, unlimited resource that can be extensively characterized with regard to ontogeny. The potential limitations of cell lines such as their restricted origin, possible *in vitro* evolution or drift, and adaptation for growth in culture, can be mitigated by use of controls that replicate culture conditions so that adaptation to *ex vivo* growth is filtered from responsive transcript lists, and by use of more than one sample. Our results show that despite the different origin, isolation, and establishment of the cell lines we used for these experiments, hemangiosarcomas retained unique characteristics that distinguished them from other cultured (or primary) cells, and that the recurrent finding of genes that are over- or under-expressed in the samples is significant and represents differences that can be traced to the developmental process of the sample (ontogeny or pathological progression), rather than to selection in culture. Ongoing experiments are designed to define the correlation of these findings in intact tumor samples where extracellular matrix associations are maintained.

Among genes whose expression differed between Golden Retrievers and non-Golden Retrievers, a disproportionately high number of genes encode transcription factors. This suggests that transcriptional regulation might play a key role in disease susceptibility and progression. Upregulation of SMARCA1 in Golden Retrievers with hemangiosarcoma was intriguing since changes in expression of a single transcriptional regulator can create genome-wide disruption of a variety of genes, possibly resulting in faster progression of the disease. It is thus feasible that deregulation of SMARCA1 potentiates susceptibility and/or heritability of hemangiosarcoma in Golden Retrievers. The downregulation of MHC class I genes in hemangiosarcoma from Golden Retrievers added a level of confidence, as these genes represent the likely targets to define individual or breed-specific differences. Preliminary assessment of MHC class I expression by flow cytometry generally support the gene expression data, with Frog (Golden Retriever) cells having no detectable MHC class I, and Dal-4 (non-Golden Retriever) cells expressing MHC class I molecules. This pattern is rather unique to hemangiosarcoma, as normal blood leukocytes and other tumors from Golden Retrievers (for example, leukemias) show robust expression of MHC class I. The organization and control of genes in the canine MHC class I locus remains poorly understood, and our data will undoubtedly spur further study of how genetic variants within breed and transforming factors might influence MHC class I expression. In fact, breed-related polymorphisms or changes in expression level have not been identified in normal canine somatic cells; thus, downregulation of MHC class I genes (at least MHC DLA-88 and DLA-64) in hemangiosarcoma cells from Golden Retrievers might reflect selective pressure to evade immune responses, or perhaps a response to autocrine or paracrine factors such as interferons or other inflammatory mediators. This illustrates the potential benefit of studies in dogs where a suitable experimental design could help distinguish whether T-cell-mediated therapies that elicit productive responses in non-Golden Retrievers might be less successful in Golden Retrievers [28], and similarly whether tumors of Golden Retrievers provide suitable targets for natural killer cell-mediated immunotherapy.

The specificity of these findings to one breed and one disease were further illustrated when we compared Golden Retrievers with hemangiosarcoma to Golden Retrievers with osteosarcoma and non-Hodgkin lymphoma. In this case, we found acid ceramidase was overexpressed in hemangiosarcomas, but not osteosarcoma or non-Hodgkin lymphoma. Acid ceramidase belongs to a family of anti-apoptotic genes that promote ceramide production. At least one inhibitor of acid ceramidases, B13, increased ceramide content selectively in tumor cells, inducing apoptosis [29], suggesting acid ceramidase inhibitors may hold therapeutic potential. It is thus possible that overexpression of this gene is a consequence of interaction among factors that underlie the observed predisposition of Golden Retrievers to hemangiosarcoma.

Another gene that was underexpressed in Golden Retrievers with hemangiosarcoma compared to non-Golden Retrievers is TSP-3, a member of the Thrombospondin family. A different member of this family, TSP-1, has potent anti-angiogenic activity [21] and has been a template for mimetics designed to treat cancer [21], [30]. Two of these mimetics, ABT-510 and ABT-526, have yielded promising results in pet dogs with a variety of tumors, albeit they were ineffective in dogs with hemangiosarcoma [31]. TSP-3 and TSP-1 are both calcium-binding proteins, but the physiological role of TSP-3 is unknown [32], [33]. The downregulation of TSP-3 should be explored further in light of these clinical results.

Despite these differences, the precise cause for increased risk to develop hemangiosarcoma in Golden Retrievers remains unclear. At least part of this perceived “risk” may be due to more rapid disease progression. In other words, it is possible that transformation of hemangiosarcoma-initiating cells does not occur with significantly greater frequency in Golden Retriever, but once it occurs, progression to clinical disease is faster, thus leading to a higher frequency of hemangiosarcoma diagnoses in Golden Retriever. An interesting correlation along these lines was the enrichment of VEGFR1 in tumors from Golden Retrievers, which generally seemed to occur at the expense of VEGFR2. It is important to note that the enrichment of VEGFR1 in tumors from Golden Retrievers was not absolute, but rather occurred in concert with various other genes that were preferentially expressed in a coordinated fashion in these cells. We tested the possibility that the “Golden Retriever background” might create a phenotype that was responsive to VEGFR1. It seemed reasonable to assume that growth of hemangiosarcoma cells, which are presumed to be of endothelial origin, was driven by VEGF. In fact, hemangiosarcoma cells make their own VEGF [23], resulting in systemic elevation of this cytokine in affected dogs [34]. The prevailing dogma states that VEGFR2 activates biochemical cascades that result in proliferation and prevent programmed cell death [35], whereas the action of VEGFR1 is less clear. VEGFR1 may transmit *bona fide* growth signals [36], [37], or it may oppose VEGFR2 signals directly or act as a decoy receptor [37], [38]. In some cases, VEGFR1 may even promote tumor growth and metastasis [36]. Our data reveal two important points. The first is that inhibition of VEGFR2 has little if any effect on proliferation of canine hemangiosarcoma cells in culture. While this may seem surprising, it is consistent with previous results in other hemangiosarcoma cell lines [39] and suggests the VEGFR2 pathway may be an ontogenic relic in these cells. That is, VEGF production and VEGFR2 expression may remain as part of the differentiation program, but the cells are not “addicted” to, or rely on, growth and survival signals transmitted through this prototypical VEGF receptor. Instead, hemangiosarcoma cells rely on other pathways for growth and survival. The second is that, at least in hemangiosarcoma cells from Golden Retrievers that express VEGFR1, this receptor may be more than simply a “decoy”, and instead, signals transmitted by VEGFR1 may dampen proliferation and/or differentiation.

These observations also are consistent with our findings that, unlike what is seen in some sporadic vascular tumors in humans, mutations of VHL are absent or infrequent in hemangiosarcoma, suggesting this disease entity may represent a distinct or specialized subset of blood vessel forming cells. Yoder et al [40] recently described a myeloid cell that is a major participant in blood vessel formation. This cell is a “vascular mimic” that can express a variety of cell surface proteins associated with endothelial precursor cells (CD133, CD34, VEGFR2), but it also has proteins that belie hematopoietic origin (CD45, CD14, CD115), has phagocytic activity, and does not contribute to the capillary endothelial layer in transplanted matrix. These findings suggest that plasticity of adult hematopoietic and mesenchymal stem cells is limited, and differentiation of myeloid progenitors into endothelial cells reflects functional rather than ontogenetic plasticity, raising the possibility that canine hemangiosarcoma is in fact a myeloid sarcoma. In this context, the inhibitory effects of VEGFR1 would be predictable, as they mirror functions of this receptor as an inhibitor of differentiation in human and murine dendritic cells. It is worth noting that enrichment for VEGFR1 and other genes may be causally related to the incidence and biological behavior of hemangiosarcoma in Golden Retrievers, but it just as likely could be an effect of other risk factors in the breed that are upstream regulators of these pathways, as our data do not distinguish between these possibilities. Nevertheless, we interpret the reproducibility of the results as an indicator that these are not simply epiphenomena.

In conclusion, our data show that gene expression profiles are informative to identify differences in tumor progression that may be influenced by heritable factors. As important, our results indicate these differences must be interpreted carefully and in the context of biological pathways. Specifically, gene expression profiling suggests that inflammation and angiogenesis are two general processes that may be sensitive to modulation by a dog's genetic background in hemangiosarcoma. Inflammation, defined by enrichment of cytokines such as IL8, IL5, IL18, and several molecules that mediate adhesion and cell-cell interactions, might reflect the action of a single aberrantly regulated molecule (for example, IL1). Angiogenesis, defined by preferential enrichment of VEGFR1 in tumor cells from Golden Retrievers might reflect engagement of unique growth (inhibitory) pathways. However, some of these differences also might reflect the ontogeny of the cells, so we must consider the possibility that the cell of origin in hemangiosarcoma retains moderate or extensive plasticity and the heritable influence is manifested based on the stage of differentiation achieved by the tumor cells. We should bear in mind, then, that part of the “susceptibility” for this disease in Golden Retrievers could be due to different biological behavior in the early stages of the disease, and also to different sensitivity of intrinsic tumor surveillance and/or chemotherapy. That is to say, upregulation of VEGFR1, downregulation of MHC class I, and downregulation of TSP-3 may underscore important differences that explain susceptibility, pathogenesis, and response to therapy. An alternative interpretation is that, regardless of the ontogeny of the tumor-initiating cell, the transformation events responsible for hemangiosarcoma involve pathways that render VEGF signals mostly inconsequential and other pathways controlled at the level of transcriptional regulation (*e.g.*, by SMARCA1) and/or survival (*e.g.*, acid ceramidase) are important determinants of the breed-dependent phenotype. Overall, this study emphasizes potential benefits of gene expression analysis and bioinformatics to study sporadic disease, and highlights the unique contribution that studies of naturally occurring cancer in man's best friend can make into disease susceptibility, heritability and progression.

## Materials and Methods

### Samples

Samples used to derive canine hemangiosarcoma cell lines from 10 pet dogs [19], [23], [24] are listed in Table 1. Only two of the dogs whose samples were used for the microarray experiments (Frog and Journey) were related within 5 generations, and they were separated by 3 generations (Frog was Journey's “great aunt”), reducing the likelihood of lineage bias. Cryopreserved cultured cells from the earliest available passages were used for these experiments. Peripheral blood samples collected from healthy dogs or from dogs with cancer prior to the initiation of any therapy (at the time of tumor biopsy) were used as controls. Non-hemangiosarcoma diagnoses included non-Hodgkin lymphoma, melanoma, and osteosarcoma. Blood samples were age and sex-matched to reduce variation. Every sample used for this study was obtained with owner consent through protocols reviewed by appropriate Institutional Animal Care and Use Committees. Samples from healthy pet dogs were obtained as part of routine diagnostic or well-health procedures. Samples from pet dogs with cancer were obtained by the attending veterinarian as part of medically necessary (biopsy) procedures or at the time of necropsy.

### RNA Isolation

RNA was isolated from tumor cells preserved in liquid nitrogen or from blood stored at −80°C using the RNAeasy Mini Kit and QIAshredder (QIAGEN, Valencia, CA), or the Ribopure Blood Kit (Ambion, Austin, TX), respectively. RNA concentration was determined using NanoDrop ND-1000 UV-Vis spectrophotometer (NanoDrop Technologies, Wilmington, DE) and quality measured using a 2100 bioanalyzer (Agilent, Santa Clara, CA).

### qPCR

Purified RNA was made into cDNA using the 1^st^ Strand cDNA Synthesis Kit for RT-PCR (Roche Applied Science, Indianapolis, IN). Real-time PCR was used to quantify cDNA using an ABI7500 sequence detector and Taqman PCR Master Mix Protocol (ABI, Foster City, CA). Each PCR was performed at 50°C for 2 min, 95°C for 10 min, and then 40 cycles of 95°C for 15 s and 60°C for 1 min per cycle. Primers and Taqman probes were designed using ABI Primer Express software (ABI). Forward primers, reverse primers, and Taqman probes (5′ to 3′ orientation) were: for DLA-88 CACCATTGTCATCGTCAGCAT, AGCTCCAATCACCCCAGAGA, and CTGCTCTGGTTCTCCT, for SMARCA-1 ATTTTGTGCATTTCATGTCTTCATC, CCTCAGCACAAGCTTCAAAGG, and AATCCTCTCAGTCCTTG, and for TSP3 TGCGAGGAGGGCGTCTT, GAGATTGGACCAAATGATGTTTTCT, and TGTATTCTGCTTCTCCC. Each PCR was done in triplicate and normalized to endogenous 18s gene using Taqman Fast Reagent Starter Kit (ABI). The samples used for real time PCR analysis (in the order presented) include CHAD-P9, Dal-4, Joey, DD1, CHAD-G6, CHAD-G4, and Frog.

### Sample Size Determination and Microarrays

Approximately 2.5 µg of RNA were labeled using the Affymetrix labeling protocol (Affymetrix, Santa Clara, CA). The cRNA samples were then hybridized to Canine_2 gene expression chips as described [41]. There are no precise tests to develop sample size estimates for gene expression profiling, so we started with theoretical principles and then applied empirical observations to support the sample size for these experiments a priori. The Canine_2.0 gene expression chip contains ∼43,000 annotated sequences derived from the 7.5× canine genome [42]. These represent virtually every known gene and a complement of expressed sequence tags that provide strong redundancy for expression profiling. We next considered that False Discovery Rate statistical analysis provided the best method to set thresholds for significance of elevated or reduced gene expression [43], but additional multivariate analyses and gene set enrichment would add further value to the analysis. We anticipated the data might not be normally distributed; so, non-parametric tests might be needed. As there is no analytical estimate of the power of the Kruskal-Wallis test after false discovery rate corrections, an approximation is useful in the case of small sample sizes. We can estimate the proportion of times when perfect rank separation between conditions might occur by chance as **2N!N!/(2N)!**., where N is the number of samples in each group [44]. Empirical tools are also available to calculate sample sizes, such as the Power Atlas (http://www.poweratlas.org, ref. [45]). Analysis of similar types of datasets in PowerAtlas suggests the sample size used for these experiments (N = 6 and 3) should provide >80% power (α = 0.05) to identify true positives, although the power to identify true negatives would be lower.

### Analysis of Gene Expression Data

Affymetrix Canine_2 microarray chip data were normalized and filtered; we used robust multiarray average (RMA) to obtain mean values for the intensity of the probe pairs and define the expression levels of the mRNA based on modeling perfect match signal intensities and ignoring mismatch signal [46]. The Canine_2 chip contains 42,900 genes; prior to statistical analysis, data were preprocessed to filter control probe sets, genes with “absent” calls in all samples, and transcripts that did not vary significantly from the median variance for the whole array. The data discussed in this publication have been deposited in NCBI's Gene Expression Omnibus [47] and are accessible through GEO Series accession number GSE15086 (http://www.ncbi.nlm.nih.gov/geo/query/acc.cgi?acc=GSE15086). After normalization and filtering, 13,758 genes remained for a comparison of Golden Retriever to non-Golden Retriever samples. There were 16 genes that differed with a *p*-value<0.001 (not corrected for multiple testing), and this list was reduced to five when corrected for multiple testing. The variation in expression for three of these was verified by qPCR. Partek software (Partek Incorporated, St. Louis, MO) was used to run analysis of variance (ANOVA) from the filtered gene lists to corroborate the gene list. These genes were ordered into hierarchical clusters using the Euclidean algorithm as the distance measure, and the Average Linkage Clustering algorithm as the linkage method, and into virtual karyotypes based on their chromosomal assignment. ONTO/express (http://vortex.cs.wayne.edu/ontoexpress/) was used to define biological function of genes from each comparison, and Gene Set Enrichment Analysis (GSEA, http://www.broad.mit.edu/gsea/) [48] to examine how expression profiles from the filtered lists fit into known and archived biological pathways.

### Immunocytochemistry and Immunoblotting

Expression of the vascular endothelial growth factor (VEGF) receptors Flk-1/VEGFR2 and Flt-1/VEGFR1 was examined by immunocytochemistry and by immunoblotting [19], [49], [50]. These experiments included an additional cell line from a Golden Retriever hemangiosarcoma (Emma) that was recently developed and therefore not used for the array experiments, but allowed us to validate gene set enrichment in an independent sample. Briefly, for immunocytochemistry cells were grown in dual chamber slides, fixed in acetone, air-dried, and stained with antibodies against VEGFR1 (Santa Cruz Biotechnology, Santa Cruz, CA) or VEGFR2 (Cell Signaling, Danvers, MA) using a modified streptavidin-biotin complex method (IHC Services, Smithville, TX). Control lysates from human umbilical vein endothelial cells (HUVEC) were purchased from Santa Cruz Biotechnology. Microscopic images were obtained using the fluorescent properties of the Fast Red dye under ultraviolet light as described [51]. Fluorescent images were acquired using an Olympus IX71 inverted microscope with an Olympus DP70 cooled digital camera (Leeds Precision Instruments, Golden Valley, MN). Transmitted light images under phase contrast were captured in automatic white balance mode. Fluorescent images were captured in automatic black balance mode (exposure times of 1/1.5 sec). Brightness for the composite image only was optimized using Adobe Photoshop CS3 (Adobe, San Jose, CA). For immunoblotting, cells were cultured to log-growth phase, dettached from plates using Accutase and extracted using RIPA buffer as described [23], [50]. Experiments to assess phosphorylation of VEGFR1 and VEGFR2 were done in cells cultured in complete media supplemented with serum and VEGF, media depleted of serum and VEGF (0.5% serum with no exogenous VEGF), or complete, supplemented media with VEGFR inhibitors “Drug 1” and “Drug 3” (see below). Inhibitors were used in experiments at a concentration range of 100 nM to 1 µM, for 30 minutes to 18 hr. Cells were harvested as described above in the presence of phosphatase inhibitors (sodium fluoride, sodium orthovanadate) and excess phosphatase substrates (sodium pyrophosphate and ß-glecrophosphate) as described [52], [53]. Modification state antibodies directed against pVEGFR1-Tyr1213 and pVEGFR2-Tyr951 were obtained from Calbiochem and Cell Signaling, and diluted for use to 1∶200 and 1∶125, respectively. Brightness and contrast for the immunoblot images were optimized using Adobe Photoshop CS3. Non-adjoining lanes (HUVEC) are demarcated by a black line.

### Cell Culture and Proliferation

The hemangiosarcoma cell lines Frog, Tucker, Dal-4, Joey, and DD-1 (Table 1) were cultured as described previously [23]. Veronica and Emma cell lines were developed as described [23] from splenic and a metastatic brain hemangiosarcomas, respectively, both from Golden Retrievers. For VEGFR inhibition, cells were cultured in the presence of small molecules that selectively inhibit VEGFR kinase (VEGF Receptor Tyrosine Kinase Inhibitor II, N-(4-Chlorophenyl)-2-[(pyridin-4-ylmethyl)amino]benzamid, hereafter called “Drug 1”; VEGFR Tyrosine Kinase Inhibitor III, KRN633, N-(2-Chloro-4-((6,7-dimethoxy-4-quinazolinyl)oxy)phenyl)-N′-propylurea, hereafter called “Drug 2”; or VEGF Receptor Kinase Inhibitor IV, 3-(3-Thienyl)-6-(4-methoxyphenyl)pyrazolo[1,5-a]pyrimidine, hereafter called “Drug 3”). The half maximal inhibitory concentrations for VEGFR1 and VEGFR2 for Drugs 1, 2, and 3, respectively are 180 and 20, 170 and 160, and 1.9 and 19. Cells (10,000/well) were plated in duplicate in 96-well microtiter plates and allowed to attach for 16 hr prior to addition of inhibitors over a concentration range from 1 pM to 1 µM. Cells were then cultured for 72 hr, and the number of viable cells was determined using the MTS assay (Promega, Madison, WI). Absorption at 490 nm for each well was averaged, and data normalized to % viability where the mean of wells that received no treatment (0 nm) was considered = 1. The results show the means of two independent experiments for each cell line.

## Supporting Information

Table S1 Complete List of 77 Gene Sets Influenced by Heritable Traits Identified Using GSEA with FDR≤0.05
(0.10 MB DOC)
