# Supplementary material for: Gene Expression Profiles of Sporadic Canine Hemangiosarcoma Are Uniquely Associated with Breed
Source: PLoS One. 2009 May 20;4(5):e5549. doi: 10.1371/journal.pone.0005549 (PMC2680013; doi:10.1371/journal.pone.0005549)
Supplement: Table S1 — Complete List of 77 Gene Sets Influenced by Heritable Traits Identified Using GSEA with FDR≤0.05 (0.10 MB DOC) [file pone.0005549.s001.doc]

**Table S1**. Complete List of 77 Gene Sets Influenced by Heritable Traits Identified Using GSEA with FDR ≤0.05

| GENE SET | ES | NES | FDR q-val |
| --- | --- | --- | --- |
| TARTE_MATURE_PC | 0.71195877 | 2.6706228 | 0 |
| IDX_TSA_DN_CLUSTER3 | 0.81700957 | 2.4078836 | 0 |
| HYPOXIA_REVIEW | 0.67453426 | 2.264447 | 0 |
| CARIES_PULP_UP | 0.73206013 | 2.2533271 | 0 |
| RUTELLA_HEPATGFSNDCS_UP | 0.69915295 | 2.1878865 | 0 |
| NAKAJIMA_MCS_UP | 0.7710549 | 2.170186 | 2.25E-04 |
| TPA_SENS_EARLY_DN | 0.6890611 | 2.1561747 | 1.93E-04 |
| BRCA_PROGNOSIS_NEG | 0.78106177 | 2.1174228 | 5.08E-04 |
| CHEN_HOXA5_TARGETS_UP | 0.76624316 | 2.1168218 | 4.52E-04 |
| ICHIBA_GVHD | 0.6342939 | 2.0915842 | 5.31E-04 |
| SHEPARD_POS_REG_OF_CELL_PROLIFERATION | 0.6963656 | 2.0740378 | 9.63E-04 |
| KNUDSEN_PMNS_UP | 0.75863785 | 2.0475783 | 0.001551264 |
| SANA_TNFA_ENDOTHELIAL_UP | 0.723751 | 2.0382078 | 0.001832481 |
| CARIES_PULP_HIGH_UP | 0.7387838 | 2.0217745 | 0.00229279 |
| VANTVEER_BREAST_OUTCOME_GOOD_VS_POOR_DN | 0.7243119 | 2.0135722 | 0.002319855 |
| IRITANI_ADPROX_VASC | 0.5824745 | 1.9871554 | 0.003367221 |
| LINDSTEDT_DEND_8H_VS_48H_UP | 0.71050835 | 1.985257 | 0.003251335 |
| BRCA_ER_NEG | 0.5130392 | 1.9838442 | 0.003070705 |
| CHIARETTI_T_ALL | 0.5994919 | 1.976144 | 0.003280133 |
| RUIZ_TENASCIN_TARGETS | 0.6596274 | 1.9748472 | 0.003255906 |
| UVC_TTD_8HR_DN | 0.64388996 | 1.9593943 | 0.004024937 |
| PASSERINI_GROWTH | 0.6465949 | 1.9592245 | 0.003841986 |
| BOQUEST_CD31PLUS_VS_CD31MINUS_UP | 0.5137528 | 1.9568404 | 0.003859861 |
| HOHENKIRK_MONOCYTE_DEND_DN | 0.68654907 | 1.9493037 | 0.00397474 |
| DIAB_NEPH_DN | 0.5628673 | 1.945688 | 0.00397444 |
| TPA_SENS_MIDDLE_DN | 0.6275479 | 1.9405124 | 0.00424034 |
| PROLIFERATION_GENES | 0.53582543 | 1.9328198 | 0.004641196 |
| BAF57_BT549_DN | 0.5604472 | 1.9226536 | 0.005634707 |
| TPA_SENS_LATE_DN | 0.6356702 | 1.9000796 | 0.007639692 |
| BRENTANI_CELL_ADHESION | 0.59833676 | 1.898688 | 0.007477455 |
| BOQUEST_CD31PLUS_VS_CD31MINUS_DN | 0.53545266 | 1.8960898 | 0.007550719 |
| PASSERINI_SIGNAL | 0.55778074 | 1.8954885 | 0.007398815 |
| GPCRS_CLASS_A_RHODOPSIN_LIKE | 0.6389767 | 1.8830441 | 0.008255412 |
| STEMCELL_COMMON_DN | 0.7151369 | 1.8819551 | 0.008213018 |
| GPCRDB_CLASS_A_RHODOPSIN_LIKE | 0.62911546 | 1.8770714 | 0.008363737 |
| LEE_DENA_UP | 0.6953906 | 1.8741663 | 0.008406119 |
| BYSTRYKH_HSC_TRANS_GLOCUS | 0.519032 | 1.872481 | 0.008317573 |
| ADIP_DIFF_CLUSTER1 | 0.703057 | 1.868296 | 0.008409626 |
| UVC_TTD_ALL_DN | 0.5802283 | 1.8654138 | 0.00850396 |
| KANG_TERT_UP | 0.69522184 | 1.8642652 | 0.008364133 |
| KUMAR_HOXA_DIFF | 0.6252044 | 1.8611827 | 0.008286815 |
| OKUMURA_MC_LPS | 0.6739458 | 1.8512058 | 0.009004677 |
| IL1_CORNEA_UP | 0.684279 | 1.8351916 | 0.010654597 |
| SASAKI_TCELL_LYMPHOMA_VS_CD4_UP | 0.59097594 | 1.8262513 | 0.011752267 |
| CELL_MOTILITY | 0.6182611 | 1.8257977 | 0.011553044 |
| UVB_NHEK3_C2 | 0.65903825 | 1.8221977 | 0.011716345 |
| MENSE_HYPOXIA_UP | 0.6587229 | 1.8033795 | 0.013945294 |
| AGED_RHESUS_UP | 0.5672658 | 1.8029629 | 0.013683071 |
| CROMER_HYPOPHARYNGEAL_MET_VS_NON_UP | 0.6567683 | 1.7932029 | 0.014337995 |
| MATSUDA_VALPHAINKT_DIFF | 0.49790433 | 1.773894 | 0.01705653 |
| SERUM_FIBROBLAST_CORE_UP | 0.6693223 | 1.7732935 | 0.016827494 |
| JAIN_NEMO_DIFF | 0.6188482 | 1.7730256 | 0.016584946 |
| IRS1_KO_ADIP_DN | 0.5519636 | 1.7551118 | 0.019020446 |
| ABRAHAM_AL_VS_MM_UP | 0.6486866 | 1.7535093 | 0.018997207 |
| RUTELLA_HEMATOGFSNDCS_DIFF | 0.45491716 | 1.7467319 | 0.019676331 |
| HIF1_TARGETS | 0.5680007 | 1.7447988 | 0.0196905 |
| SASAKI_ATL_UP | 0.59097594 | 1.7444369 | 0.019345054 |
| CHIARETTI_T_ALL_DIFF | 0.5138973 | 1.7364756 | 0.020650752 |
| AGEING_KIDNEY_UP | 0.51945746 | 1.7261546 | 0.022538383 |
| IL6_FIBRO_UP | 0.65780354 | 1.72582 | 0.022316393 |
| CMV_HCMV_TIMECOURSE_ALL_DN | 0.4964983 | 1.7148678 | 0.024417829 |
| CELL_ADHESION | 0.53694457 | 1.7066474 | 0.026101882 |
| VEGF_MMMEC_ALL_UP | 0.5801034 | 1.7045926 | 0.026304211 |
| GALINDO_ACT_UP | 0.59468293 | 1.7023925 | 0.026483174 |
| EGF_HDMEC_UP | 0.6437848 | 1.6968653 | 0.027484085 |
| GH_EXOGENOUS_ANY_DN | 0.5999015 | 1.6872257 | 0.029308813 |
| ADDYA_K562_HEMIN_TREATMENT | 0.6080544 | 1.684632 | 0.029481564 |
| ROS_MOUSE_AORTA_DN | 0.6276916 | 1.6695524 | 0.03379748 |
| VEGF_MMMEC_6HRS_UP | 0.57878184 | 1.6670033 | 0.03393805 |
| UVC_TTD_4HR_DN | 0.56942725 | 1.6655447 | 0.03372668 |
| LEE_CIP_UP | 0.56914234 | 1.6648719 | 0.033502206 |
| HOFMANN_MANTEL_LYMPHOMA_VS_LYMPH_NODES_UP | 0.6199711 | 1.6607134 | 0.03421717 |
| RADMACHER_AMLNORMALKARYTYPE_SIG | 0.6224322 | 1.6466782 | 0.037877336 |
| CHANG_SERUM_RESPONSE_UP | 0.5809775 | 1.6465735 | 0.037386607 |
| NI2_MOUSE_UP | 0.62013805 | 1.6325101 | 0.04126826 |
| HOHENKIRK_MONOCYTE_DEND_UP | 0.54141444 | 1.6221558 | 0.04457358 |
| AGEING_KIDNEY_SPECIFIC_UP | 0.52963257 | 1.6117783 | 0.047980957 |
| TENEDINI_MEGAKARYOCYTIC_GENES | 0.54281485 | 1.5969602 | 0.052990574 |
